# Propranolol for Paroxysmal Sympathetic Hyperactivity with Lateralizing Hyperhidrosis after Stroke

**DOI:** 10.1155/2015/421563

**Published:** 2015-06-18

**Authors:** Jason W. Siefferman, George Lai

**Affiliations:** ^1^Department of Neurology, VA New York Harbor Healthcare System, 423 East 23rd Street No. 127, New York, NY 10010, USA; ^2^Department of Neurology, Mount Sinai Beth Israel Medical Center, New York, NY 10003, USA

## Abstract

Brain injury can lead to impaired cortical inhibition of the hypothalamus, resulting in increased sympathetic nervous system activation. Symptoms of paroxysmal sympathetic hyperactivity may include hyperthermia, tachycardia, tachypnea, vasodilation, and hyperhidrosis. We report the case of a 41-year-old man who suffered from a left middle cerebral artery stroke and subsequently developed central fever, contralateral temperature change, and hyperhidrosis. His symptoms abated with low-dose propranolol and then returned upon discontinuation. Restarting propranolol again stopped his symptoms. This represents the first report of propranolol being used for unilateral dysautonomia after stroke. Propranolol is a lipophilic nonselective beta-blocker which easily crosses the blood-brain barrier and may be used to treat paroxysmal sympathetic hyperactivity.

## 1. Introduction

Stroke, among other causes of brain injury, may impair central autonomic regulatory centers leading to development of tachycardia, hyperhidrosis, hypertension, and hyperthermia. Although the parasympathetic system may also be involved, sympathetic overactivity is more common [1]. These symptoms resulting from autonomic dysregulation are collectively referred to as dysautonomia or paroxysmal sympathetic hyperactivity (PSH) [2], and stroke represents the third most common cause [3].

Although the pathophysiology of PSH is poorly understood, the dominant theories currently revolve around failures of the central autonomic network (CAN), including the insular cortex, amygdala, hypothalamus, medulla, periaqueductal gray matter, parabrachial complex, and nucleus of the tractus solitarius. With stroke, PSH has been attributed to damage to the insular cortex, amygdala, and hypothalamus, which mediate high-order autonomic controls [4]. The loss of cortical inhibition of the periventricular hypothalamus activates the sympathetic nervous system and results in increased contralateral sympathetic outflow. Although a variety of agents have been used to manage PSH [5, 6], we report the first case of contralateral PSH after ischemic stroke which was treated successfully with propranolol.

## 2. Case Presentation

A 41-year-old man was admitted for acute rehabilitation after a right middle cerebral artery ischemic stroke which was not treated with a thrombolytic agent. His past medical history included poorly controlled hypertension, type 2 diabetes mellitus (DM), and prior transient ischemic attacks. He had no known allergies to medications and denied having had surgeries.

Eight days after the stroke, he complained of a cold sensation and excessive perspiration on the right side of his face and body. He also began having daily fevers (max 101.5°F) associated with tachycardia (max 119 beats per minute). He had no significant tachypnea (range 18–20 breaths per minute) or hypertension (max systolic blood pressure 132 mm Hg, max diastolic 83 mm Hg), although he was taking fosinopril. Extensive workup revealed no infectious source, and his fever did not abate with repeated doses of acetaminophen.

He was fully conscious, alert, and oriented at each examination and fully participating in physical therapy. On inspection, excessive sweating was noted along the right side of his body and lower right side of his face. On palpation, his right arm was cool to the touch. Neurologic examination revealed normal mental status and cranial nerves II–XII were intact. Strength testing revealed right upper extremity strength of 4/5 for shoulder abduction, elbow flexion, elbow extension, and handgrip. The right lower extremity exhibited 3/5 strength for hip flexion, 4/5 for knee flexion, and 2/5 for ankle dorsiflexion. Tone was diffusely increased on the right, rated 1/4 on the modified Ashworth scale. Reflexes were 2+ and symmetrical in the biceps brachioradialis, triceps, and gastrocnemius-soleus complex; however, the right great toe was upgoing. Sensation was impaired to temperature and light touch along the right side of his body and face.

Magnetic resonance imaging (MRI) revealed a large area of infarction in the left periventricular white matter, left internal capsule posterior limb, and dorsal aspect of left lateral basal ganglia extending into white matter adjacent to the left occipital horn.

With a presumptive diagnosis of PSH, propranolol 10 mg twice daily was started. After demonstrating tolerance and persistent breakthrough symptoms, dosing was increased to three times daily. The patient's symptoms of unilateral cold sensations, hyperhidrosis, tachycardia, and fever resolved within one day. As a trial, propranolol was discontinued, and, although no fever or tachycardia was documented, symptoms of unilateral hyperhidrosis, cold sensations, and subjective fever returned. Propranolol was then restarted, and the patient continued to have symptomatic relief at follow-up.

Using the PSH Assessment Measure [2], our patient scored 4/18 on the Clinical Features Scale and 9/11 on the diagnosis Likelihood Tool. The total score of 13 suggests the diagnosis of PSH is “possible” (range 8–16). He had no identifiable triggers for his symptoms, such as an allodynic reaction, and only the fevers and tachycardia were paroxysmal; the hyperhidrosis and cold sensation on the right side were constant. Aside from the spasticity attributed to his stroke, he experienced no dystonic posturing associated with paroxysms. Unlike many patients with traumatic brain injury, our patient had a focal unilateral ischemic stroke, which likely tempered the severity of his symptoms in comparison to those with more severe or bilateral injuries.

## 3. Discussion

Normally, the efferent sympathetic sudomotor and vasomotor pathways which originate in the hypothalamus and descend into the brainstem uncrossed receive inhibitory input from the contralateral insular cortex [4, 7]. Infarction of this pathway or of the hypothalamus can lead to unopposed contralateral sympathetic outflow. Dysautonomia, or PSH, is a relatively common disorder which is a frequent complication of ischemic stroke and commonly manifests as cardiovascular or sudomotor dysregulation [8]. The importance of recognizing and treating these cases is supported by the finding that a majority of these patients will suffer poor prognoses involving severe disability and death [3, 9]. Unfortunately, there is lack of literature demonstrating pathophysiology and defining management.

Several central autonomic regulatory centers have been implicated in various disconnection theories. Korpelainen et al. noted that frontoparietal cortex, hypothalamus, and brainstem injuries in stroke patients resulted in tachycardia, flushed skin, hyperhidrosis, and cutaneous sensory changes [10]. Another study by Naver et al. found that 16 of 37 stroke patients experienced cold sensations associated with decreased basal skin blood flow and temperature contralateral to the side of the lesion [11]. In fact, Korpelainen et al. demonstrated that central autonomic network damage can lead to skin cooling up to 6.8°C in the contralateral limb [8]. Feedback to the hypothalamus from the cooled side may trigger a systemic increase of the core body temperature. Friedman and Naver later suggested that such thermal sensatory changes are due to loss of inhibitory signals from the left hemisphere, since no patients with right cortical hemispheric lesions had experienced cold sensations in Naver's study [12]. Our patient also suffered a left-sided stroke and experienced right-sided limb cooling.

More recently, Meyer et al. proposed that autonomic control could be lateralized to the right insular cortex, as injury there led to more severe and frequent autonomic symptoms such as hyperhidrosis [13]. Sweating is physiologically controlled by 2 centers in the hypothalamus and limbic systems which control thermoregulation and emotional sweating, respectively [14]. Hyperhidrosis is a relatively common complaint in patients with hemispheric stroke and dysautonomia [15]. Two case reports presented patients with infarcts localized to the opercular cortex and hypothalamus who experienced contralateral hyperhidrosis lasting days to months [16, 17]. In fact, the degree of hyperhidrosis has been shown to correlate with the severity of paresis and presence of pyramidal tract signs of hemispheric stroke [15].

Although our patient experienced relatively common PSH symptoms, he also experienced an unusual daily fever. Paroxysmal persistent hyperthermia, although rare in occurrence, can be due to various intracranial etiologies but is usually associated with injury of the hypothalamus in poststroke patients [18]. Although our patient's hypothalamus was not affected by his stroke, he still experienced persistent hyperthermia with no infectious etiology found on workup. In 2007, Oh et al. presented a patient with a brain neoplasm and subsequent cerebral hemorrhage who experienced paroxysmal persistent hyperthermia that did not respond to antibiotics or steroids [19]. This patient's hyperthermia did, however, respond to propranolol.

In our patient, propranolol 10 mg three times daily was effective in alleviating not only his fever but also his unilateral hyperhidrosis, tachycardia, and cold sensations. Propranolol is a nonselective beta-blocker which is lipophilic and easily crosses the blood-brain barrier. It has been FDA-approved for treatment of hypertension, angina pectoris due to coronary artery atherosclerosis, migraine, hypertrophic subaortic stenosis, tachyarrhythmias, and certain tremors. It has also been noted for its use in PSH [20]. Other medications reported for treating PSH include opiates, dopamine agonists, benzodiazepines, baclofen, and gabapentin [5, 6].

## 4. Conclusion

Hemispheric stroke may impair autonomic regulation resulting in paroxysmal sympathetic hyperactivity, which may present with central fever, tachycardia, tachypnea, hypertension, hyperhidrosis, dystonic posturing, and sympathetic overreactivity to nonpainful stimuli. Our case demonstrated the successful use of propranolol in controlling symptoms of fever, tachycardia, unilateral hyperhidrosis, and coldness. The precise effect that propranolol has on the impaired central autonomic network and autonomic nervous system has yet to be elucidated, and this warrants further investigation.
